# Deficiency for the ER-stress transducer OASIS causes severe recessive osteogenesis imperfecta in humans

**DOI:** 10.1186/1750-1172-8-154

**Published:** 2013-09-30

**Authors:** Sofie Symoens, Fransiska Malfait, Sanne D’hondt, Bert Callewaert, Annelies Dheedene, Wouter Steyaert, Hans Peter Bächinger, Anne De Paepe, Hulya Kayserili, Paul J Coucke

**Affiliations:** 1 Center for Medical Genetics, Ghent University Hospital, 9000 Ghent, Belgium; 2 Research Department, Shriners Hospitals for Children, 97239 Portland, OR, USA; 3 Department of Biochemistry and Molecular Biology, Oregon Health & Science University, 97239 Portland, OR, USA; 4 Department of Medical Genetics, Istanbul Medical Faculty, Istanbul University, 34093 Istanbul, Turkey

**Keywords:** Osteogenesis imperfecta, Type I collagen, OASIS, CREB3L1, Endoplasmic reticulum stress

## Abstract

Osteogenesis imperfecta (OI) is a clinically and genetically heterogeneous brittle bone disorder. Whereas dominant OI is mostly due to heterozygous mutations in either *COL1A1* or *COL1A2*, encoding type I procollagen, recessive OI is caused by biallelic mutations in genes encoding proteins involved in type I procollagen processing or chaperoning. Hitherto, some OI cases remain molecularly unexplained. We detected a homozygous genomic deletion of *CREB3L1* in a family with severe OI. *CREB3L1* encodes OASIS, an endoplasmic reticulum-stress transducer that regulates type I procollagen expression during murine bone formation. This is the first report linking *CREB3L1* to human recessive OI, thereby expanding the OI gene spectrum.

## Background

Osteogenesis imperfecta (OI) is a genetically heterogeneous brittle bone disorder with varying degrees of clinical severity, ranging from perinatal lethality to generalized osteopenia [1]. The predominant autosomal dominant forms display mutations in either *COL1A1* or *COL1A2*, encoding the α1- and α2-chains of type I procollagen, while rarer autosomal recessive forms mostly result from defective endoplasmic reticulum (ER)-resident proteins involved in post-translational processing or chaperoning of these α(I)-chains [1,2]. Processing defects prevent normal collagen fibrillogenesis and on biochemical analysis often show perturbed modification of the collagen α-chain. Known defects include biallelic mutations in *LEPRE1*[3-5], *CRTAP*[5,6], *PPIB*[7,8], *BMP1*[9,10], and *PLOD2*[11]. Mutations in chaperones (including Hsp47 (*SERPINH1*) and FKBP10) impair intracellular collagen trafficking with intracellular retention or aggregation of collagen molecules and show dilation of the ER on electron microscopy, resulting in OI or related phenotypes [12-14]. Finally, rare other defects linked to distinct mechanisms involve the transcription factor osterix (*SP7*) [15], pigment epithelium derived factor (*SERPINF1*) [16] and transmembrane protein 38B (*TMEM38B*) [17,18]. A recurrent mutation in a gene encoding the Interferon-inducible transmembrane protein 5 (*IFITM5*), which is involved in bone growth during prenatal murine development, was recently shown to cause autosomal (AD) dominant OI [19-21]. Recently, heterozygous and homozygous mutations in *WNT1* (WNT1), which is a key signalling molecule in osteoblast function and bone development, were shown to underlie certain forms of AD early-onset osteoporosis and AR OI, which was in some patients associated with severe intellectual disability [22-26]. However, a small proportion of OI patients remain molecularly unexplained.

## Findings

**Figure 1:**
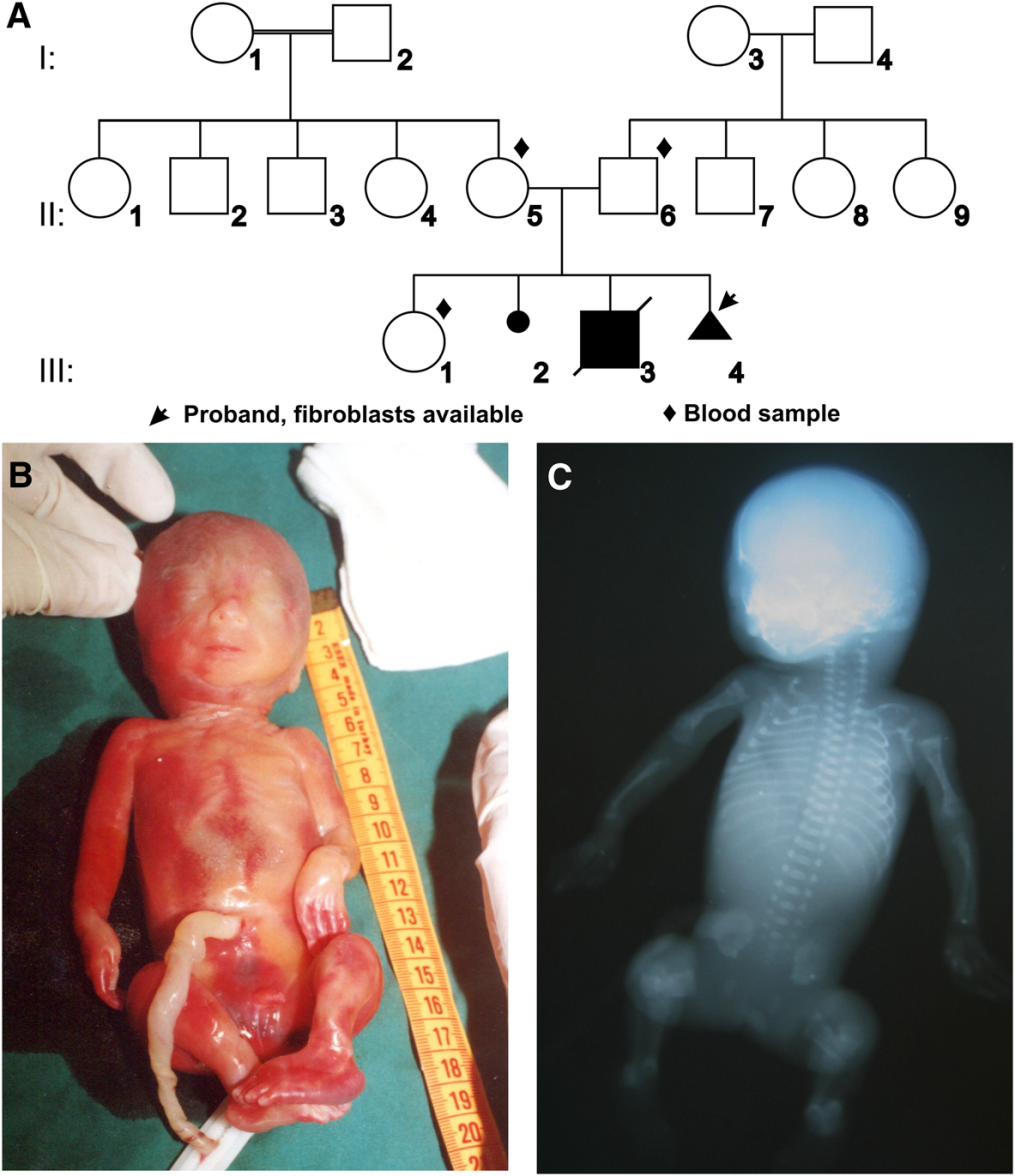
**Pedigree and clinical findings. A**. Pedigree of the Turkish family. **B**. Post-mortem examination of foetus III:4 at 19 weeks of gestation showed bowed extremities and pes equinovarus. **C**. X-rays of foetus III:4 revealed beaded ribs and multiple fractures of tubular bones.

We describe a Turkish family (Figure 1A) with three sibs, two of whom were affected by severe OI (written informed consent of the family was obtained and the study was approved by the Ethics Committee of the Ghent University Hospital (Ghent, Belgium)). Consanguinity was not reported, but the parents originated from neighbouring villages. The first affected child (III:3) developed several fractures *in utero* and was small for gestational age. His birth length was 40 cm (<P3). At the first day of life he was hospitalized for hyperbilirubinemia and O-bain-like deformities, soft calvarial bones and widely open fontanelles were noticed. He developed several fractures after birth and multiple fractures healed with extremity deformities. He also had a right inguinal hernia. X-rays showed beaded ribs, callus formation and multiple fractured tubular bones with an accordion-like broadened appearance. He was hospitalized several times due to recurrent constipation and pulmonary infections (bronchopneumonia). During this period, he developed abdominal distention and hepatomegaly, the latter due to cardiac insufficiency. No signs of T-cell dysfunction or other immune deficiencies have been noted. He died at 9 months of age. The second affected sib (III:4, Figure 1A) was a male foetus from a pregnancy that was medically terminated at 19 weeks of gestation. Post-mortem examination showed thin ribs and fractures at bowed humerus and femora (Figure 1B-C).

The parents have a healthy daughter (III:1) and have had one miscarriage (III:2, cause unknown). The adolescent daughter has blue sclerae but had not experienced any fractures. The mother (II:5) at 38 years of age and the father (II:6) at 47 years have blue sclerae, a soft and velvety skin and normal teeth. While the mother has small joint hypermobility, the father has conductive hearing loss.

**Figure 2:**
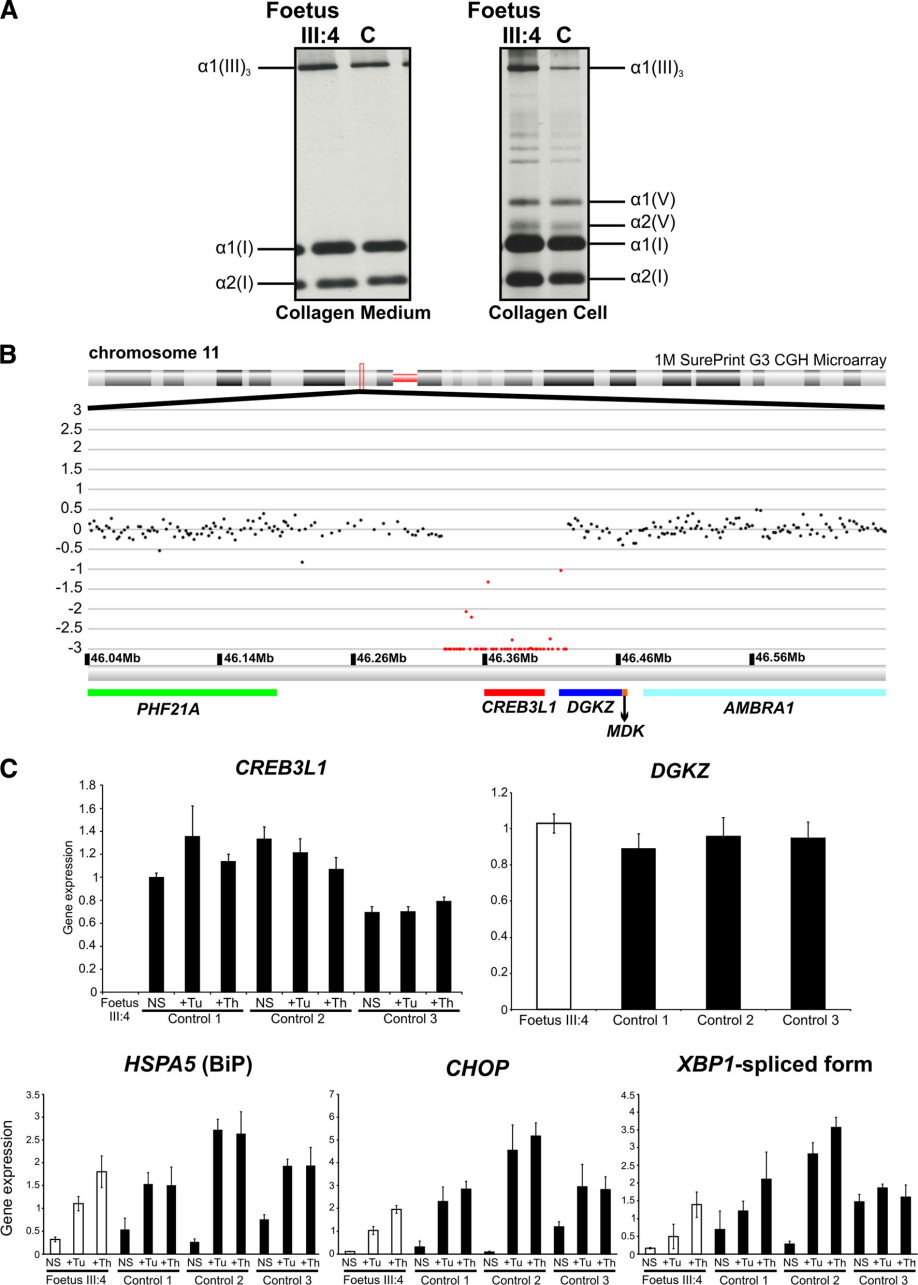
**Biochemical and molecular results. A**. Biochemical collagen analysis was performed on collagens produced by the patients dermal fibroblasts, which were grown for 16 hrs in the presence of ^14^C-Proline. Radioactively labelled intracellular and secreted fibrillar collagen proteins were isolated and mature collagens were obtained by pepsin digestion. Foetal secreted (left panel) as well as intracellular (right panel) mature type I collagen revealed a normal electrophoretic pattern when compared to a control (C) sample. Also for the unprocessed, secreted type I procollagen a normal electrophoretic migration pattern was observed (data not shown). **B**. ArrayCGH analysis on a 1M SurePrint G3 Human CGH Microarray revealed a homozygous deletion of the entire *CREB3L1* gene in the affected foetus III:4. **C**. Expression level analysis by RT-qPCR was performed in duplicate on total RNA extracted from three biological replicates of the fibroblast cell lines from foetus III:4 and three controls (C1, C2 and C3) (LightCycler480 and RealTime ready DNA Probe Master Mix, Roche). The expression level of each investigated gene was quantified using qbase^PLUS^ (Biogazelle) [27]. *HPRT1*, *RLP13a* and *YWHAZ* were applied as reference targets. RT-qPCR for foetus III:4 confirmed the total absence of *CREB3L1* expression when compared to control samples (C1, C2 and C3). DGKZ has two alternative (tissue-specific) isoforms [28].

Biochemical (pro)collagen SDS-PAGE analysis was performed on the medium and cellular fractions of cultured skin fibroblasts of foetus III:4. No obvious quantitative or qualitative abnormalities of ^14^C-labelled type I procollagen (data not shown) and mature secreted and intracellular type I collagen (Figure 2A) were detected.

Subsequently, all known OI genes (*COL1A1*, *COL1A2*, *BMP1*, *LEPRE1*, *CRTAP*, *PPIB*, *PLOD2*, *SERPINH1*, *FKBP10*, *SP7*, *SERPINF1*, *TMEM38B, IFITM5*and *WNT1*) were sequenced by direct Sanger sequencing (ABI3730XL automated sequencer, Applied Biosystems), but no causal mutation(s) were detected.

We selected the *CREB3L1* gene [GenBank:NM_052854.2], encoding the ER-stress transducer OASIS (Old Astrocyte Specifically Induced Substance), as an excellent candidate gene based on the observation that OASIS-/- mice were born with severe osteopenia and spontaneous fractures [29], reminiscent of severe human OI. In those mice, OASIS was shown to be crucial for bone formation through activating *col1a1* transcription and facilitating the secretion of matrix proteins. Treatment of murine osteoblasts with BMP-2 (bone morphogenic protein 2) causes mild ER-stress and is associated with accelerated RIP (regulated intramembrane proteolysis) of OASIS. The N-terminal part of OASIS is subsequently translocated to the nucleus, where it binds to the osteoblast-specific UPRE (unfolded protein response element) regulatory region in the murine *Col1a1* promoter thereby causing high levels of type I procollagen expression [29]. While the amount of type I procollagen is normal in the murine OASIS-/- skin, reduced amounts of type I procollagen were detected in OASIS-/- calvaria and tibia, which suggested tissue-specific decrease of type I procollagen in the bone matrix but also failure of the OASIS-/- osteoblasts to produce high levels of type I procollagen [29]. OASIS further functions as a tissue-specific ER-stress transducer that alters transcription of target genes involved in developmental processes, differentiation, or maturation upon mild ER-stress. PCR amplification of all exons and flanking introns of *CREB3L1* failed in foetus III:4, suggesting a homozygous whole gene deletion. ArrayCGH analysis (1M SurePrint G3 Human CGH Microarray, Agilent Technologies) and copy number profiling (arrayCGHbase) confirmed this genomic deletion, which encompasses *CREB3L1* and the first exon of *DGKZ* (arr11p11.2(46268141–46359490)×0, Figure 2B) [30,31]. Whereas the arr11p11.2(46268141–46359490)×0 homozygous deletion was not reported before, heterozygous deletions or gains of this genomic region are described in the Decipher database [32] and the Database of Genomic Variants [33] but encompassing large genomic regions comprising multiple genes (6 to 86 genes and/or multiple chromosomal abnormalities) which, in some cases, are associated with intellectual disability. Both parents and the healthy sister were heterozygous for the deletion (data not shown). *DGKZ* encodes diacylglycerol kinase zeta, an ubiquitously expressed enzyme that is most abundantly present in the brain, thymus and skeletal muscle [34] and which has a regulatory role in T-cell receptor signalling and T-cell activation [35]. Two different isoforms (DGKζ1 in immune cells and DGKζ2 in other cells) are known, in which exon 1 is either present or absent and which have a tissue- and developmental stage-specific expression [28]. Hitherto, no known function in bone formation has been ascribed to DGKζ and thus a possible contributing role to (the severity of) the bone phenotype of patient III:3 and foetus III:4 cannot completely be excluded. Expression analysis by real time-quantitative PCR (RT-qPCR) on total RNA isolated from dermal fibroblasts of foetus III:4 confirmed complete absence of the *CREB3L1* transcript. In order to investigate the expression of the two DGKZ isoforms (DGKζ1 and DGKζ2), two different primer pairs were designed, of which one was specific for exon 1 that is only present in the DGKζ1 isoform. RT-qPCR experiments revealed no amplification for the primer pair specific for exon 1 in cultured dermal fibroblasts, suggesting that the DGKζ1 isoform is not expressed in these cells. For the second primer pair normal DGKZ expression was observed, which implies normal expression of the DGKζ2 isoform in cultured human dermal fibroblasts (Figure 2C). RT-qPCR analysis of the ER-stress markers BiP, CHOP and the spliced form of XBP1 showed levels comparable to controls, even after stimulation of confluent fibroblasts for 4 hours with the ER-stress inducers Tunicamycin (Tu, 10 μg/ml, Sigma-Aldrich) and Thapsigargin (Th, 1 μM, Sigma-Aldrich) (Figure 2C). This is in accordance to the observations in OASIS-/- mice. The expression level of *CREB3L1* was unchanged in control fibroblasts after treatment with Tu and Th (Figure 2C), suggesting that OASIS does not play a major role in the ER-stress pathways previously linked to disease pathogenesis [1]. Additionally, our finding that type I (pro)collagen production is normal in human dermal fibroblasts (Figure 2A) confirms that OASIS has a tissue-specific effect on type I (pro)collagen production [29].

In conclusion, the identification of *CREB3L1* (encoding the ER-stress transducer OASIS) as a novel gene for autosomal recessive OI expands the spectrum of genes linked to OI and reinforces the role of ER-stress in the pathophysiology of OI.

## Competing interests

The authors declare that they have no competing interests.

## Authors’ contributions

Conceived and designed the experiments: SS, PC. Identified and recruited human subjects, obtained ethical approvals, coordinated collection of samples, and provided clinical information: FM, BC, HK, ADP. Performed the experiments: SS, SD, AD, WS. Analyzed the data: SS, PC. Wrote the paper: SS, FM, BC, ADP, PC. All authors have read and approved the final manuscript.
